# Galangin Induces Autophagy *via* Deacetylation of LC3 by SIRT1 in HepG2 Cells

**DOI:** 10.1038/srep30496

**Published:** 2016-07-27

**Authors:** Xv Li, Yajun Wang, Yuzhen Xiong, Jun Wu, Hang Ding, Xiaoyi Chen, Liubo Lan, Haitao Zhang

**Affiliations:** 1 Department of Biochemistry and Molecular Biology, Guangdong Medical University, Zhanjiang, Guangdong 524023, China; 2 Affiliated Hospital of Guangdong Medical University, Zhanjiang, Guangdong 524023, China

## Abstract

Galangin suppresses proliferation and induces apoptosis and autophagy in hepatocellular carcinoma (HCC) cells, but the precise mechanism is not clear. In this study, we demonstrated that galangin induced autophagy, enhanced the binding of SIRT1-LC3 and reduced the acetylation of endogenous LC3 in HepG2 cells. But this autophagy was inhibited by inactivation of SIRT1 meanwhile, galangin failed to reduce the acetylation of endogenous LC3 after SIRT1 was knocked-down. Collectively, these findings demonstrate a new mechanism by which galangin induces autophagy *via* the deacetylation of endogenous LC3 by SIRT1.

Galangin (3,5,7-trihydroxyflavone) is a flavonoid derived primarily from the rhizome *Alpinia officinarum* (Hance). It is used in traditional Chinese medicine and is contained in some foods^1^. Galangin likely undergoes oxidative metabolism mainly in the liver^2^. Galangin inhibits proliferation, induces apoptosis and promotes autophagy in hepatocellular carcinoma cells^3^,^4^,^5^,^6^,^7^,^8^, but the precise mechanism by which galangin induces autophagy remains unclear.

Autophagy is a self-digestion process, during which cytoplasmic components, such as protein aggregates, damaged organelles, and lipid vesicles, are sequestered into double-membraned structures called autophagosomes. Autophagosomes then fuse with lysosomes for subsequent degradation of the content^9^,^10^,^11^,^12^.

The microtubule-associated protein 1 light chain 3 (LC3) is a key regulator of autophagy that controls major steps in the autophagic pathway, such as the growth of autophagic membranes, the recognition of autophagic cargoes, and the fusion of autophagosomes with lysosomes^13^,^14^,^15^,^16^. The conversion of LC3 I to LC3 II *via* proteolytic cleavage and lipidation is a mark of mammalian autophagy^12^,^17^. Sequestosome 1 (p62/SQSTM1), which is degraded by autophagy, is another indicator of autophagy^18^,^19^. Other products of autophagy-related genes (ATGs), such as ATG3 and Beclin1 (also called ATG6/Vps30), also perform essential roles in autophagy^20^,^21^. Recent research has shown that the deacetylation of LC3, ATG5 and ATG7 by mammalian silent information regulator 2 homolog 1 (SIRT1) is essential for autophagosome formation under starvation conditions^22^,^23^.

SIRT1 is an NAD-dependent class III histone deacetylase that plays major roles in regulating gene expression, DNA damage repair, metabolism, tumor development, aging, and autophagy^22^,^23^,^24^,^25^,^26^. SIRT1 promotes autophagy through the adenosine monophosphate-activated protein kinase (AMPK)/SIRT1 signaling pathway^27^ or *via* deacetylation of ATG5, ATG7, ATG8 (LC3)^22^,^23^ or forkhead box O1 (FOXO1)^28^.

In this study, we investigated the autophagy-inducing effect of galangin in HepG2 cells and explored the mechanism by which galangin induces autophagy *via* the deacetylation of endogenous LC3 by SIRT1.

## Methods

### Cell culture

The human liver cancer cell line HepG2 was obtained from the American Type Culture Collection (Rockville, Md., USA) and maintained at the Institute of Biochemistry and Molecular Biology at Guangdong Medical College. This cell line was cultured in Dulbecco’s modified Eagle’s medium (DMEM, Gibco BRL) supplemented with 10% fetal bovine serum (Sijiqing Laboratories, Hangzhou, China), 100 mg/mL penicillin, and 100 mg/mL streptomycin. The cells were incubated at 37 °C in a humidified atmosphere containing 5% CO_2_.

### Agents and chemicals

Galangin (PubChem CID: 5281616) was purchased from Sigma–Aldrich and dissolved in dimethyl sulfoxide (DMSO) before addition to the cell cultures. The final concentration of DMSO in the culture medium was kept below 0.1% (v/v) after the addition of galangin. TSA was purchased from Tokyo Chemical Industry. Rabbit or mouse polyclonal antibodies against Beclin1, LC3, acetylated lysine, and GAPDH were purchased from Cell Signaling Technology. Rabbit or mouse polyclonal antibodies against SIRT1 and p62 were purchased from Abcam. A rabbit polyclonal antibody against β-actin was purchased from Beijing Biosynthesis Biotechnology.

### Autophagy induction

To induce autophagy, HepG2 cells were treated with 130 μM galangin for 24 hours. For the co-immunoprecipitation experiments, HepG2 cells were treated with 130 μM galangin for 18 hours to induce autophagy. We repeated the experiments three times.

### Transmission electron microscope

HepG2 cells were harvested by trypsinization, then washed twice with PBS, fixed with the buffer containing 3% glutaraldehyde and 0.1 M cacodylate, and then re-fixed in osmium tetroxide. HepG2 cells were embedded in Epong and cut into sections with a thickness of 1.0 μm. Sections were stained with methylene buffer ArumeII and then viewed with a Philips electron microscope CM-120.

### Inactivating SIRT1 and regulating its expression

EX-527 is a specific SIRT1 inhibitor that inhibits SIRT1 activity at a concentration of 50 μM^22^. Therefore, we treated HepG2 cells with 50 μM EX-527 for 2 hours to inhibit SIRT1 activity, induced autophagy by adding 130 μM galangin into the medium containing EX-527 and continued to cultivate the cells for 24 hours.

To regulate the expression of SIRT1, HepG2 cells were infected with relevant recombinant adenoviruses (Shanghai GeneChem Co., Ltd.) for 2 hours and then cultured in fresh complete medium for 24 hours. Next, the cells were treated with 130 μM galangin to induce autophagy. We repeated these experiments three times.

### Detection of autophagy using GFP-LC3

The green fluorescent protein and LC3 (GFP-LC3) fusion protein provides a useful indicator of autophagy initiation through the evaluation of LC3 dots or punctae. When autophagy occurs, GFP-LC3 foci redistribute from a diffuse pattern to a punctate cytoplasmic pattern (GFP-LC3 punctae), and the percentage of cells with GFP-LC3 punctae increases^29^.

HepG2 cells were transfected with ptfLC3 (Addgene, Plasmid #21074), a highly specific fluorescent marker of autophagy, to measure autophagy levels. Lipofectamine^®^ 3000 Reagent (Life Technologies) was used to transfect HepG2 cells. After the induction of autophagy with 130 μM galangin, the cellular localization of GFP-LC3 was visualized using a Nikon fluorescence microscope. We repeated these experiments three times.

### Western blot and co-immunoprecipitation analysis

Western blot analysis was performed using whole cell extracts prepared by lysing the cells in lysis buffer (pH 8.0) containing 50 mM Tris–HCl, 150 mM NaCl, 5 mM EDTA, 1% NP40, 0.05% PMSF, 2 mg/mL aprotinin, and 2 mg/mL leupeptin. Antibodies against GAPDH and β-actin were used to assess the purity of the respective fractions.

For the co-immunoprecipitation analyses, the cells were resuspended in Nonidet P40 lysis buffer containing 10 mM nicotinamide and 10 μM TSA. Cell lysates were mixed with antibodies at 4 °C overnight, and PureProteome™ Protein A/G Mix Magnetic Beads (Millipore Corporation) were then added. The detailed protocol for this procedure can be found at www.millipore.com. The proteins were resolved by SDS-polyacrylamide gel electrophoresis (PAGE) and then transferred onto polyvinylidene fluoride membranes, which were blocked in 5% (w/v) nonfat milk and hybridized with specific primary antibodies. The resulting protein bands were visualized using ECL (Thermo Scientific) after hybridization with a secondary antibody (Thermo Scientific). All of the western blot results were quantified using Image J. We repeated these experiments three times.

## Results

### Galangin induced autophagy and upregulated SIRT1 expression in HepG2 cells

**Figure 1:**
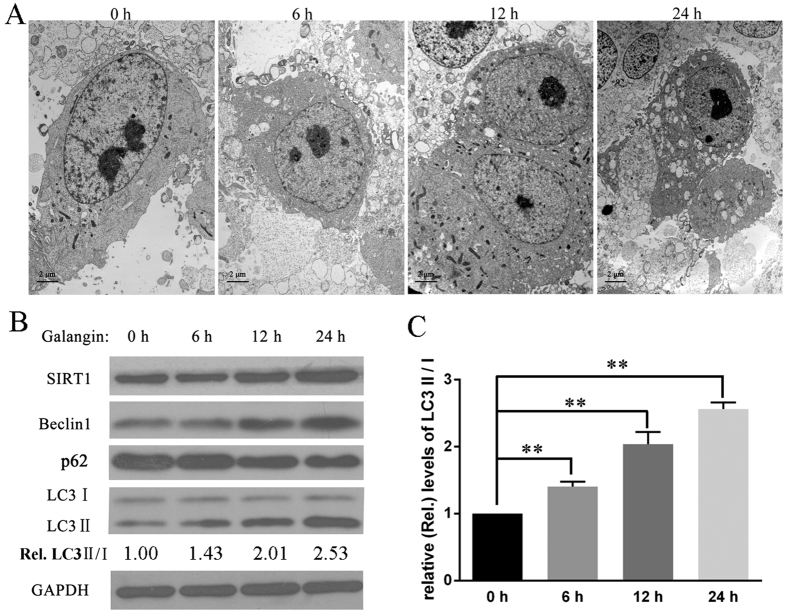
Galangin induced autophagy and upregulated SIRT1 expression in HepG2 cells. (**A**) HepG2 cells were treated with 130 μM galangin for 0, 6, 12 and 24 hours, then visualized by transmission electron microscopy (×5800). (**B**) The expression of SIRT1, Beclin1, p62 and LC3 were detected by western blot. (**C**) The relative (Rel.) levels of LC3 II/ LC3 I, **P < 0.01, *n* = 3.

Our previous study demonstrated that 130 μM galangin induces autophagy in hepatocellular carcinoma cells^5^,^7^,^8^. In this study, we treated HepG2 cells with 130 μM galangin and then detected autophagy by transmission electron microscopy and western blot analysis. Results showed that galangin promoted the appearance of autophagic vacuoles, increased the expression of LC3 II, Beclin1, and the ratio of LC3 II to LC3 I and decreased the expression of p62 in a time-dependent manner (Fig. 1). The autophagy-inducing effects of galangin were consistent with those in our previous study^5^,^7^,^8^. During the induction of autophagy, galangin increased SIRT1 expression in a time-dependent manner (Fig. 1B).

### EX-527 inhibited SIRT1 and Galangin-induced autophagy

**Figure 2:**
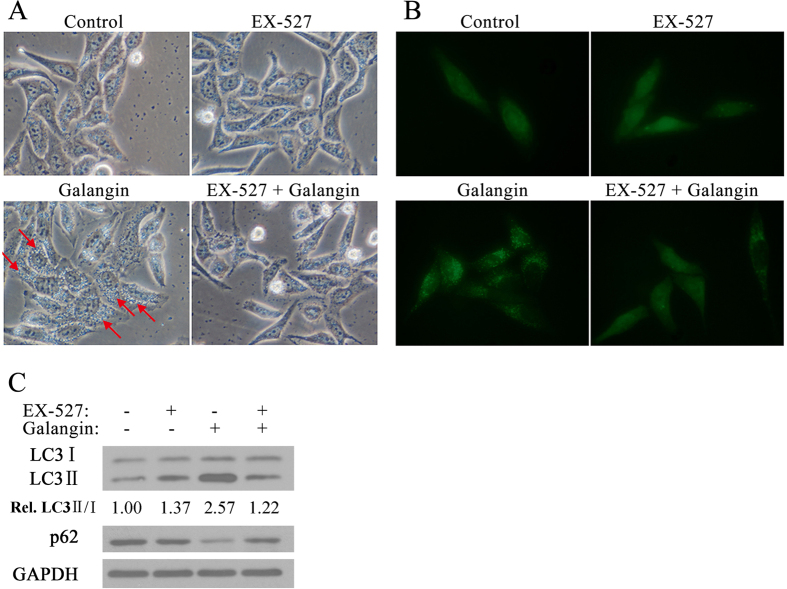
EX-527 inhibited SIRT1 and Galangin-induced autophagy. (**A**) Autophagic vacuolization (indicated by red arrows) in HepG2 cells treated with EX-527 and galangin. Cells were visualized using a phase contrast microscope (×200). (**B**) Distribution of GFP-LC3 in HepG2 cells treated with EX-527 and galangin. Cells were transfected with GFP-LC3 exposed to different treatment conditions, and visualized by fluorescent microscopy (×200). (**C**) LC3 and p62 expression in HepG2 cells exposed to 50 μM EX-527 and 130 μM galangin.

To confirm that SIRT1 participates in galangin-induced autophagy, we pretreated HepG2 cells with the specific SIRT1 inhibitor EX-527 (50 μM) for 2 hours, then, cells were treated with 130 μM galangin for 24 hours. Results showed that EX-527 inhibited galangin-induced autophagic vacuolization (Fig. 2A) and GFP-LC3 punctae formation (Fig. 2B). EX-527 also blocked the conversion of endogenous LC3 I to LC3 II and restored the galangin-induced downregulation of p62 (Fig. 2C). These data suggest that galangin-induced autophagy is inhibited by EX-527 in HepG2 cells.

### SIRT1 is essential to Galangin-induced autophagy

**Figure 3:**
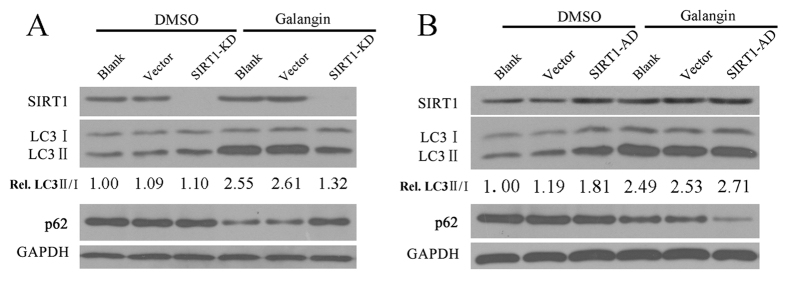
SIRT1 is essential to Galangin-induced autophagy. (**A**) LC3 and p62 expression in DMSO treated or 130 μM galangin-treated HepG2 cells under following conditions: uninfected control (Blank), SIRT1-knockdown (SIRT1-KD), vector-infected control for SIRT1-KD (Vector). (**B**) LC3 and p62 expression in DMSO treated or 130 μM galangin-treated HepG2 cells under following conditions: uninfected control (Blank), SIRT1 over-expression (SIRT1-AD) and vector-infected control for SIRT1-AD (Vector).

We infected HepG2 cells with relevant recombinant adenoviruses to knockdown or upregulate SIRT1. Then, cells were treated with 130 μM galangin for 24 hours to induce autophagy. Compared to vector-infected cells (Vector) and uninfected cells (Blank), the conversion of LC3 I to LC3 II and downregulation of p62 was blocked in SIRT1-knockdown cells treated with galangin (Fig. 3A), indicating that galangin-induced autophagy was inhibited by SIRT1 downregulation. In addition, overexpressing SIRT1 increased the conversion of LC3 I to LC3 II and reduced the expression of p62, indicating that overexpressing SIRT1 stimulated basal autophagy of HepG2 cells under normal fed condition (Fig. 3B).

### Deacetylation of endogenous LC3 by SIRT1 was essential for galangin-induced autophagy

**Figure 4:**
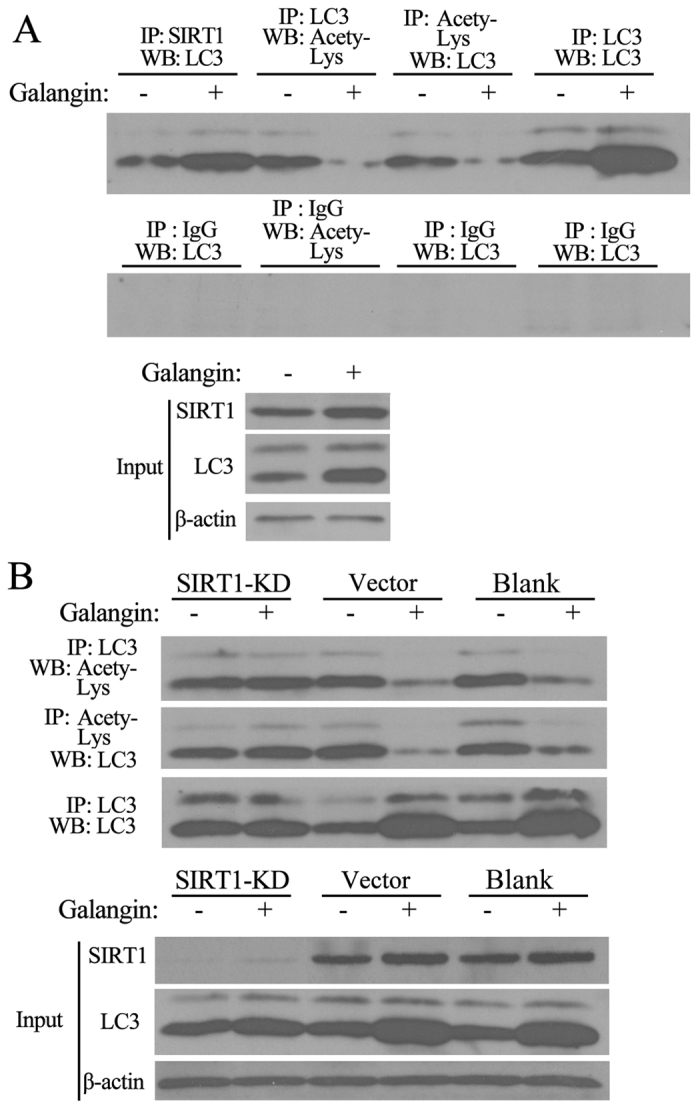
Deacetylation of endogenous LC3 by SIRT1 was essential for galangin-induced autophagy. (**A**) The binding of endogenous SIRT1 to endogenous LC3 and endogenous LC3 acetylation were detected using co-immunoprecipitation assays and western blot analysis in HepG2 cells treated with DMSO or 130 μM galangin for 18 hours. (**B**) Endogenous LC3 acetylation was detected by co-immunoprecipitation assays and western blot analysis after HepG2 cells were treated with DMSO or 130 μM galangin in SIRT1-KD, Vector, and uninfected cells (Blank).

To determine the mechanism by which SIRT1 promotes galangin-induced autophagy, we used co-immunoprecipitation assays to detect the binding of endogenous SIRT1 and LC3. Results showed that the binding of SIRT1 and LC3 was enhanced and the acetylation of endogenous LC3 was reduced after HepG2 cells were treated with 130 μM galangin for 18 hours (Fig. 4A).

To further explore whether endogenous LC3 was deacetylated by SIRT1 in galangin-induced autophagy, we knocked down SIRT1 expression in HepG2 cells and then treated cells with galangin for 18 hours. In vector-infected cells and uninfected cells, galangin promoted the conversion of LC3 I to LC3 II and decreased the acetylation of LC3. But in SIRT1-knockdown cells, galangin failed to reduce the acetylation of LC3 (Fig. 4B). These results suggest that deacetylation of endogenous LC3 by SIRT1 is essential for galangin-induced autophagy in HepG2 cells.

In conclusion, this study demonstrates a new mechanism by which galangin induces autophagy *via* the deacetylation of endogenous LC3 by SIRT1 in HepG2 cells (Fig. 4).

## Discussion

Many flavonoids, such as quercetin, sudachitin, kaempferol, and luteolin, have been reported to upregulate SIRT1 expression or activate SIRT1^30^,^31^,^32^,^33^. Furthermore, certain flavonoids, such as catechin, epicatechin, quercetin, and myricetin, can induce autophagy^34^. However, the role of SIRT1 in flavonoid-induced autophagy had not been reported. In this study, we showed that SIRT1 expression was upregulated in a time-dependent manner in HepG2 cells during galangin-induced autophagy, which was inhibited when SIRT1 was inactivated or knocked down. In addition, overexpressing SIRT1 stimulated basal autophagy of HepG2 cells. Our data suggest that galangin, which has a simple flavonoid structure, can induce autophagy and upregulate SIRT1 in HepG2 cells and that SIRT1 is essential for galangin-induced autophagy. This study provides a connection between flavonoids, autophagy and SIRT1.

Then, we explored the mechanism by which SIRT1 regulates galangin-induced autophagy. Researches have demonstrated the relationship between SIRT1 and autophagy; for example, SIRT1 regulates autophagy *via* the deacetylation of several ATGs^22^,^23^ and FoxO1^28^ and *via* the AMPK/SIRT1 signaling pathway^27^. However, the mechanism by which SIRT1 influences flavonoid-induced autophagy had not been reported. ATGs perform important roles in autophagy, and the deacetylation of ATG5, ATG7 and ATG8 (LC3) by SIRT1 is necessary for the induction of starvation-induced autophagy^22^,^23^. Thus, we speculated that SIRT1 may participate in galangin-induced autophagy through deacetylation of ATG8 (LC3). In this study, we demonstrated that LC3-SIRT1 binding and endogenous LC3 deacetylation by SIRT1 play important roles in galangin-induced autophagy. These results provide a new mechanism by which galangin induces autophagy *via* the deacetylation of endogenous LC3 by SIRT1 in HepG2 cells.

**Figure 5:**
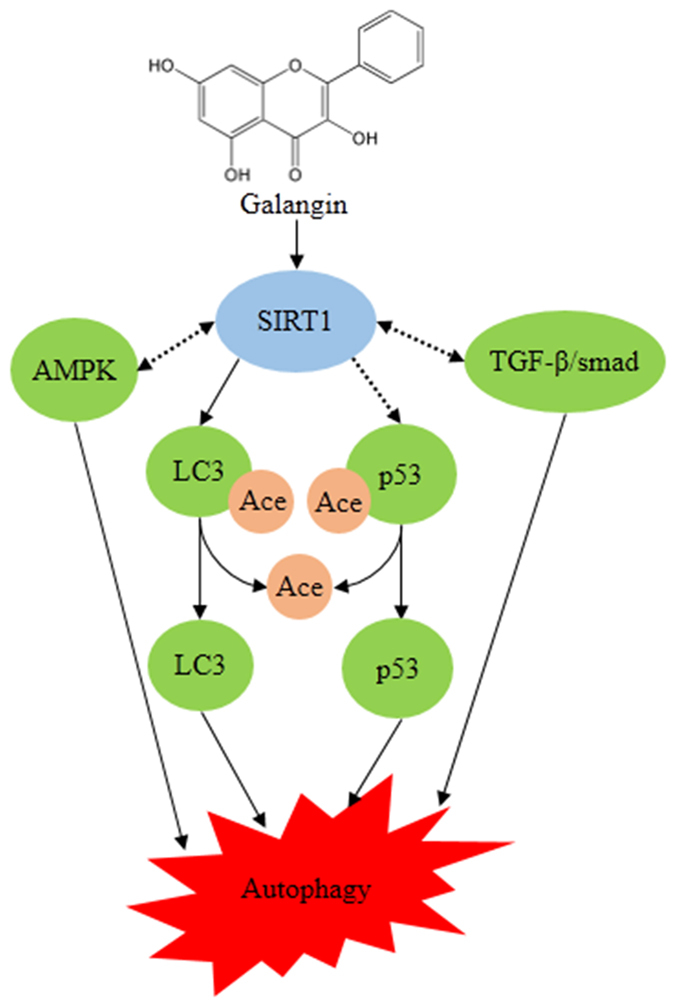
The autophagy-inducing effect of galangin in HCC. The actual lines indicate that the pathway has been demonstrated. The dotted lines indicate that the pathway is needed to be explored.

Based on previous experimental results, we demonstrate the mechanisms by which galangin induces autophagy as shown in Fig. 5. Galangin induces autophagy in hepatocellular carcinoma cells by activating the TGF-β receptor/Smad pathway^8^, by activating AMPK *via* increasing the AMP/TAN ratio^7^, by upregulating p53^5^ and by deacetylating LC3. All these pathways have been reported to share a close relationship with SIRT1. AMPK is a key energy sensors of the cell. Simultaneously, SIRT1/AMPK signaling pathway has been reported to induce autophagy in oxidative stress^35^, glucose starvation^36^, chronic colitis^37^ and resveratrol treatment^38^,^39^,^40^. So galangin may induce glucose starvation, and then activiates SIRT1/AMPK pathway, leads to autophagy finally. Further more, the deacetylation of p53 by SIRT1 is essential for capsaicin induced autophagy^41^. Zerr *et al*. have reported that SIRT1 can activate TGF-β/Smad pathway^42^. And then Smad3 also in turn activates SIRT1 transcription^43^. Basing on our results that galangin-induced autophagy was block by inactiviating or down-regulating SIRT1, we think that the autophagy-inducing effect of galangin may depend on SIRT1. As many flavonoids can activiate SIRT1 and induce aotuphagy, we believe that some kind of flavonoids may induce autophagy mainly through SIRT1.

Autophagy is a highly conserved process that involves degradation and recycling of proteins and organelles to generate nucleotides, amino acids, fatty acids, sugars, and ATP to support metabolism and survival under adverse microenvironmental conditions^11^,^44^. Autophagy is thought to play dual roles in cancer because it enables the survival of tumor cells by promoting metabolite turnover and absorption, inhibiting apoptosis and reactive oxygen species production, and increasing drug resistance^45^; in contrast, it can prevent tumor initiation by suppressing chronic tissue damage, inflammation, and genome instability^46^, and many studies have shown that autophagy induction can contribute to apoptosis^47^,^48^,^49^. Similarly, SIRT1 has opposing effects in cancer. On the one hand, SIRT1 inactivates tumor suppressors, activates proto-oncogenes, and promotes cancer cell proliferation, invasion, migration, and chemoresistance, which confer a survival advantage to cancer cells^50^,^51^,^52^,^53^,^54^,^55^,^56^. On the other hand, SIRT1 suppresses tumors by inhibiting inflammation and the activity of transcription factors that exacerbate carcinogenesis and by preserving genomic stability^57^,^58^,^59^,^60^,^61^,^62^. In our study, galangin inhibited the growth of HepG2 cells mainly by potently inducing continuous autophagy, thereby inhibiting cell proliferation and inducing apoptosis^7^,^8^. Here, we demonstrated that SIRT1 is essential for galangin-induced autophagy, suggesting that SIRT1 and autophagy play an anti-cancer role under the treatment of galangin.

These findings suggest that the SIRT1-activating and autophagy-inducing functions of flavonoids are worth further exploration for cancer therapy, as are the autophagy-regulating functions of SIRT1. In conclusion, galangin induces autophagy *via* the deacetylation of LC3 by SIRT1 in HepG2 cells. Combined with the results of our previous reports, the current data suggest that galangin is a potential anti-cancer drug.

## Additional Information

**How to cite this article**: Li, X. *et al*. Galangin Induces Autophagy *via* Deacetylation of LC3 by SIRT1 in HepG2 Cells. *Sci. Rep*. **6**, 30496; doi: 10.1038/srep30496 (2016).
